# Supplementary material for: Generative Adversarial Phonology: Modeling Unsupervised Phonetic and Phonological Learning With Neural Networks
Source: Front Artif Intell. 2020 Jul 8;3:44. doi: 10.3389/frai.2020.00044 (PMC7861218; doi:10.3389/frai.2020.00044)
Supplement: Supplementary file 1 [file Data_Sheet_1.pdf]

Supplementary Material: Beguš, Generative Adversarial Phonology

1 TRAINING DATA: GAMMA REGRESSION

To confirm the presence of the durational distribution in VOT between the #TV and #sTV sequences in the training data, VOT durations based on TIMIT's manual annotations were measured across the two conditions. VOT in TIMIT is annotated from the release of the stop to the onset of the following vowel. Slices for which no VOT duration exists (only closure duration that includes the VOT) were excluded from this analysis, but were included in the training; altogether 47 sequences were thus excluded. While the TIMIT database is occasionally misaligned, the errors are minor and likely do not crucially affect the outcomes. Table 1 and Figure 3 (in the paper) summarize raw VOT durations across three places of articulation. Speaker identity is not included in the model, because it is irrelevant for the purpose of training a GAN network.

To test the significance of the presence of [s] as a predictor of VOT duration, the data were fit to a Gamma regression model (with log-link) with two predictors: STRUCTURE (the presence vs. absence of [s]) and PLACE of articulation of the target stop (with three levels — [p], [t], [k]) and their interaction. STRUCTURE was treatment-coded (with absence of [s] as the reference level), while PLACE of articulation of the stop was sum-coded (with [k] as reference). The interaction term is significant (AIC = 46873.25 vs. 46885.73), which is why it is kept in the final model. The model (see Table S1) shows that at the mean of the PLACE of articulation as a predictor, VOT is significantly shorter if T is preceded by [s] ($\beta = -0.84, t = -49.69, p < 0.0001$). Fitted values for #TV are 56.97 ms [56.41, 57.53] ms and for #sTV 24.62 ms [23.86, 25.41]. The difference between the means is 32.35 ms. The ratio of VOT durations (estimated with the *emmeans* package; Lenth 2018) between the two conditions ($\frac{\#TV}{\#sTV}$) equals 2.34, (SE = 0.039). Figure 3 (in the paper) illustrates the significant difference and its magnitude between the two conditions across the three places of articulation. The significant interaction #sTV:[t] is not informative for our purposes.

	Estimate	Std. Error	t value	Pr(>|t|)
(Intercept)	4.0426	0.0050	804.3778	0.0000
#TV vs. #sTV	-0.8389	0.0169	-49.6883	0.0000
[p] vs. mean	-0.1383	0.0079	-17.5160	0.0000
[t] vs. mean	-0.0311	0.0068	-4.5706	0.0000
#sTV:[p]	-0.1026	0.0257	-3.9979	0.0001
#sTV:[t]	0.0694	0.0214	3.2432	0.0012

Table S1. Coefficients of a Gamma regression model with duration of VOT in the training data as the dependent variable and condition (#TV vs. #sTV) and PLACE of articulation (with interaction) as independent variables.

2 GENERATED DATA: GAMMA REGRESSION

To test the significance of the observed distribution in the generated data, the data were fit to a Gamma regression model with VOT duration as the dependent variable and only one predictor: the presence of [s] (STRUCTURE). Place of articulation and following vowel were not added in the model, because they

are often difficult to recover. STRUCTURE is a significant predictor of VOT duration: $\beta = -0.46, t = -9.17, p < 0.0001$[1]. Fitted values for #TV are 61.47 ms with 95% CI [57.33, 65.91] and for #sTV 38.68 ms with 95% CI [36.06, 41.50]. The difference between the means is 22.79 ms. The ratio of VOT durations (estimated with *emmeans* package; Lenth 2018) between the two conditions ($\frac{\#TV}{\#sTV}$) equals 1.59 (SE = 0.080).

3 LATENT VARIABLES: LASSO REGRESSION AND RANDOM FOREST MODELS

To further test the accuracy of the regression model (in Section 4.3.1 in the paper) in identifying the variables that correlate with [s] in the output, the data were also fit to Lasso regression and Random Forest models. The same seven variables are also identified as having the highest estimates in a Lasso regression for binomial data, estimated with the *glmnet* package (Simon et al., 2011) with cross-validated lambda values. Almost identical results are also derived with the Balanced Random Forest approach (estimated in *randomForest* package in Liaw and Wiener 2002). The seven variables have the highest Mean decrease Gini estimates in a random forest model after 2,500 trees and with 9 variables randomly sampled for each tree. There is again a substantial decrease in estimates after the seven values. Mean decrease accuracy gives a similar ranking, with the exception that z_5 is the 8th highest predictor and z_{74} the 18th highest. The accuracy of this estimate is highly variable with the choice of number of variables sampled and likely not as accurate as the regression models (possibly due to the fact that error rate for the presence of [s] group is high in the model — 74.2%). The value of variables were chosen based on smallest OOB error rate (tried on a range from 9 to 15 with 2,500 trees). We sample 271 variables from each group (the presence vs. absence of [s]) each time to correct for the unbalanced sample.

4 GENERATIVE TEST 2

The proportions from the Generative test 2 (Section 4.3.3 in the paper) were fit to a beta regression linear model[2] (using *mgcv* package; Wood 2011). The independent variables are estimates of the regression models in Figure 10 (in the paper) for each of the 31 variables tested. In fact, we can test which of the six regression models (from generalized additive to linear logistic regression) makes the best predictions about the latent variables and the correlation of the variables with the presence of [s] in the output. Six models were fit, one for each of the six regression models presented (FULL, SELECT, MODIFIED, EXCLUDED, LINEAR, LINEAREXCLUDED). The best-fitting model was chosen based on AIC: estimates of z-variables in the LINEAR model (Figure 10 in the paper) make the best predictions regarding the presence or absence of [s] in the output as tested with this independent generative approach.

5 INTERPOLATION

Number of knots for the model in Table S3 is chosen as the default in the smooth term and as 5 in the random smooths. There is negative autocorrelation at lag 1, but with so little variance left unexplained (99.5%; adjusted $R^2 = 0.99$), this likely does not affect outcomes substantially (Sóskuthy, 2017). Autocorrelation is reduced when the ratio is modeled as normally distributed and correction for AR(1) correlation is added to the model with $\rho = 0.98$ (Baayen et al., 2016). This, however, introduces a substantially worse fit. Since estimates of the smoothing terms are similar (with the same smooths being significant), we keep the beta regression model with autocorrelation.

[1] Estimates for Intercept (when no [s] precedes) are $\beta = -2.79, t = -78.34, p < 0.0001$.

[2] Generalized additive models do not provide a better fit and in none of the six models is a smooth significantly different from a linear line.

		SELECT		
A. parametric coef.	Estimate	Std. Error	t-value	p-value
(Intercept)	-5.3046	0.2104	-25.2179	< 0.0001
B. smooth terms	edf	Ref.df	F-value	p-value
s(z_5)	0.9828	9.0	57.0935	0.0000
s(z_{11})	0.9823	9.0	55.4790	0.0000
s(z_{14})	0.9791	9.0	46.7389	0.0000
s(z_{26})	0.9802	9.0	49.5906	0.0000
s(z_{29})	1.6222	9.0	51.2550	0.0000
s(z_{49})	0.9819	9.0	54.1608	0.0000
s(z_{74})	2.3630	9.0	50.3333	0.0000
		LINEAR EXCLUDED		
	Estimate	Std. Error	z-value	Pr(>|z|)
(Intercept)	-6.1378	0.2879	-21.32	0.0000
z_5	1.3678	0.1770	7.73	0.0000
z_{11}	-1.3619	0.1725	-7.89	0.0000
z_{14}	1.2739	0.1759	7.24	0.0000
z_{26}	1.2932	0.1725	7.50	0.0000
z_{29}	-1.3234	0.1705	-7.76	0.0000
z_{49}	-1.3557	0.1747	-7.76	0.0000
z_{74}	-1.3280	0.1795	-7.40	0.0000

Table S2. Coefficients of the seven predictors with highest χ^2 values or highest slope estimates from two models: SELECT and LINEAR EXCLUDED.

A. parametric coefficients	Estimate	Std. Error	t-value	p-value
(Intercept) = z_{11}	-0.0571	0.0156	-3.6505	0.0003
z_5	-0.0404	0.0123	-3.2820	0.0011
z_{14}	-0.0011	0.0144	-0.0753	0.9400
z_{26}	-0.0097	0.0115	-0.8444	0.3989
z_{29}	-0.0590	0.0113	-5.2131	< 0.0001
z_{49}	0.0074	0.0112	0.6595	0.5100
z_{74}	-0.0741	0.0121	-6.1071	< 0.0001
B. smooth terms	edf	Ref.df	F-value	p-value
s(zValuePerc):z_5	1.0002	1.0000	11.8417	0.0006
s(zValuePerc):z_{11}	4.1696	4.8546	14.1190	< 0.0001
s(zValuePerc):z_{14}	5.3322	6.1117	36.6899	< 0.0001
s(zValuePerc):z_{26}	1.0003	1.0002	12.5952	0.0004
s(zValuePerc):z_{29}	1.0002	1.0000	12.0036	0.0006
s(zValuePerc):z_{49}	4.2002	4.8650	19.1225	< 0.0001
s(zValuePerc):z_{74}	3.2768	3.7863	1.2326	0.2479
fs(zValuePerc,sameValues,m=1,k=5)	110.6863	143.0000	6.1542	< 0.0001
fs(zValuePerc,trajectoryZ,m=1,k=5)	558.2060	728.0000	56.9670	< 0.0001

Table S3. Coefficients of a beta regression generalized additive model with ratio of maximum intensity ([s] vs. [s] + vowel) as the dependent variable.

A. parametric coefficients	Estimate	Std. Error	t-value	p-value
(Intercept) = z_{11}	4751.7378	84.7008	56.1002	< 0.0001
z_5	218.6576	116.9490	1.8697	0.0618
z_{14}	-236.4061	134.6301	-1.7560	0.0793
z_{26}	195.2722	108.9736	1.7919	0.0734
z_{29}	103.6866	107.8602	0.9613	0.3366
z_{49}	17.6464	106.4109	0.1658	0.8683
z_{74}	108.7466	113.8531	0.9551	0.3397
**B. smooth terms**	**edf**	**Ref.df**	**F-value**	**p-value**
s(zValuePerc) = z_{11}	7.5348	7.9933	12.1238	< 0.0001
s(zValuePerc):z_5	4.5539	5.7457	2.9261	0.0081
s(zValuePerc):z_{14}	7.3604	8.3734	5.7228	< 0.0001
s(zValuePerc):z_{26}	5.5900	6.8683	3.8049	0.0005
s(zValuePerc):z_{29}	5.8536	7.1301	2.9198	0.0045
s(zValuePerc):z_{49}	4.4714	5.6434	1.8590	0.0803
s(zValuePerc):z_{74}	4.2765	5.4186	2.8162	0.0136
fs(zValuePerc,sameValues,m=1,k=10)	143.4989	288.0000	1.0560	< 0.0001
fs(zValuePerc,trajectoryZ,m=1,k=7)	168.3558	1120.0000	0.2032	< 0.0001

Table S4. Coefficients of a generalized additive model with center of gravity as the dependent variable with the marginal value of z-variables (STRONG). The model was fit with correction for autocorrelation with $\rho = 0.7$.

A. parametric coefficients	Estimate	Std. Error	t-value	p-value
(Intercept) = z_{11}	1.0675	0.1045	10.2167	< 0.0001
z_5	-0.4521	0.1420	-3.1842	0.0015
z_{14}	0.3405	0.1693	2.0105	0.0446
z_{26}	-0.4434	0.1323	-3.3517	0.0008
z_{29}	-0.6225	0.1339	-4.6502	< 0.0001
z_{49}	0.0431	0.1332	0.3234	0.7464
z_{74}	-0.5129	0.1383	-3.7077	0.0002
**B. smooth terms**	**edf**	**Ref.df**	**F-value**	**p-value**
s(zValuePerc) = z_{11}	3.3590	4.0455	4.4859	0.0013
s(zValuePerc):z_5	1.0001	1.0001	1.8978	0.1686
s(zValuePerc):z_{14}	5.7086	6.9165	2.9066	0.0054
s(zValuePerc):z_{26}	1.0000	1.0000	2.3717	0.1238
s(zValuePerc):z_{29}	2.2995	2.8348	1.4855	0.2361
s(zValuePerc):z_{49}	5.3523	6.5335	2.5656	0.0106
s(zValuePerc):z_{74}	1.0000	1.0000	0.1912	0.6620
fs(zValuePerc,sameValues,m=1,k=10)	69.5866	288.0000	0.4214	< 0.0001
fs(zValuePerc,trajectoryZ,m=1,k=7)	174.7382	1120.0000	0.2422	< 0.0001

Table S5. Coefficients of a generalized additive model with kurtosis as the dependent variable with the marginal value of z-variables (STRONG). The model was fit with correction for autocorrelation with $\rho = 0.2$.

A. parametric coefficients	Estimate	Std. Error	t-value	p-value
(Intercept) = z_{11}	0.2726	0.0841	3.2434	0.0012
z_5	-0.2686	0.1197	-2.2448	0.0249
z_{14}	-0.0188	0.1377	-0.1368	0.8912
z_{26}	-0.1965	0.1115	-1.7629	0.0781
z_{29}	-0.2011	0.1101	-1.8270	0.0679
z_{49}	-0.0403	0.1063	-0.3792	0.7046
z_{74}	-0.2468	0.1165	-2.1193	0.0342
B. smooth terms	edf	Ref.df	F-value	p-value
s(zValuePerc) = z_{11}	4.5857	5.4215	1.3591	0.3433
s(zValuePerc):z_5	4.2885	5.5104	2.0917	0.0864
s(zValuePerc):z_{14}	6.4497	7.7372	3.3262	0.0009
s(zValuePerc):z_{26}	6.4653	7.7452	2.2045	0.0303
s(zValuePerc):z_{29}	3.8520	4.9849	2.0158	0.0716
s(zValuePerc):z_{49}	1.0000	1.0001	0.0105	0.9186
s(zValuePerc):z_{74}	4.0239	5.1943	1.9009	0.0916
fs(zValuePerc,sameValues,m=1,k=10)	113.6068	288.0000	0.6943	< 0.0001
fs(zValuePerc,trajectoryZ,m=1,k=7)	0.0001	1120.0000	0.0000	0.9908

Table S6. Coefficients of a generalized additive model with skew as the dependent variable with the marginal value of z-variables (STRONG). The model was fit with correction for autocorrelation with $\rho = 0.7$.

A. parametric coefficients	Estimate	Std. Error	t-value	p-value
(Intercept) = z_{11}	4396.2895	88.8182	49.4976	< 0.0001
z_5	2.4059	85.3386	0.0282	0.9775
z_{14}	109.3881	101.5196	1.0775	0.2815
z_{26}	98.1943	79.3503	1.2375	0.2162
z_{29}	-34.1064	78.4139	-0.4350	0.6637
z_{49}	-42.5635	77.1872	-0.5514	0.5815
z_{74}	19.5268	85.7248	0.2278	0.8199
B. smooth terms	edf	Ref.df	F-value	p-value
s(zValuePerc) = z_{11}	6.9763	7.4135	16.1815	< 0.0001
s(zValuePerc):z_5	1.0002	1.0003	0.0007	0.9793
s(zValuePerc):z_{14}	2.1327	2.4962	1.0245	0.2793
s(zValuePerc):z_{26}	1.0072	1.0110	0.2178	0.6468
s(zValuePerc):z_{29}	1.0003	1.0004	0.4048	0.5249
s(zValuePerc):z_{49}	1.0001	1.0002	3.5280	0.0606
s(zValuePerc):z_{74}	2.4946	2.9500	1.2008	0.2568
fs(zValuePerc,sameValues,m=1,k=10)	198.6356	288.0000	3.3009	< 0.0001
fs(zValuePerc,trajectoryZ,m=1,k=7)	413.0783	1120.0000	1.6191	< 0.0001

Table S7. Coefficients of a generalized additive model with center of gravity as the dependent variable with the value of z-variables at the point before [s] ceases from the output (WEAK).

A. parametric coefficients	Estimate	Std. Error	t-value	p-value
(Intercept) = z_{11}	0.6420	0.1544	4.1575	< 0.0001
z_5	-0.0037	0.1463	-0.0256	0.9796
z_{14}	-0.4010	0.1685	-2.3803	0.0174
z_{26}	0.0230	0.1368	0.1678	0.8668
z_{29}	0.0909	0.1344	0.6762	0.4991
z_{49}	0.0870	0.1323	0.6576	0.5109
z_{74}	0.1577	0.1429	1.1032	0.2702
B. smooth terms	edf	Ref.df	F-value	p-value
s(zValuePerc) = z_{11}	2.6481	2.9134	1.5639	0.2107
s(zValuePerc):z_5	1.0000	1.0000	3.2961	0.0697
s(zValuePerc):z_{14}	1.0000	1.0001	0.5569	0.4556
s(zValuePerc):z_{26}	1.9006	2.3489	1.0773	0.3078
s(zValuePerc):z_{29}	1.0000	1.0000	0.0284	0.8661
s(zValuePerc):z_{49}	1.0000	1.0000	0.0002	0.9887
s(zValuePerc):z_{74}	1.3675	1.6177	0.3165	0.5648
fs(zValuePerc,sameValues,m=1,k=10)	181.3987	288.0000	2.3885	< 0.0001
fs(zValuePerc,trajectoryZ,m=1,k=7)	128.9479	1120.0000	0.1673	< 0.0001

Table S8. Coefficients of a generalized additive model with kurtosis as the dependent variable with the value of z-variables at the point before [s] ceases from the output (WEAK). The model was fit with correction for autocorrelation with $\rho = 0.2$.

A. parametric coefficients	Estimate	Std. Error	t-value	p-value
(Intercept) = z_{11}	0.2432	0.0734	3.3145	0.0009
z_5	-0.0384	0.0758	-0.5067	0.6125
z_{14}	-0.0906	0.0873	-1.0373	0.2998
z_{26}	-0.1433	0.0705	-2.0325	0.0423
z_{29}	-0.0392	0.0698	-0.5613	0.5747
z_{49}	-0.0191	0.0687	-0.2777	0.7813
z_{74}	-0.0151	0.0740	-0.2043	0.8381
B. smooth terms	edf	Ref.df	F-value	p-value
s(zValuePerc) = z_{11}	5.2698	5.9125	3.3712	0.0037
s(zValuePerc):z_5	1.0000	1.0000	0.5871	0.4437
s(zValuePerc):z_{14}	1.0000	1.0000	1.7508	0.1860
s(zValuePerc):z_{26}	1.0000	1.0000	0.1276	0.7210
s(zValuePerc):z_{29}	1.5340	1.8995	0.3881	0.6718
s(zValuePerc):z_{49}	1.0000	1.0000	0.5952	0.4406
s(zValuePerc):z_{74}	2.1616	2.7315	0.7639	0.3898
fs(zValuePerc,sameValues,m=1,k=10)	170.1493	288.0000	2.2288	< 0.0001
fs(zValuePerc,trajectoryZ,m=1,k=7)	47.7523	1120.0000	0.0661	0.0001

Table S9. Coefficients of a generalized additive model with skew as the dependent variable with the value of z-variables at the point before [s] ceases from the output (WEAK). The model was fit with correction for autocorrelation with $\rho = 0.3$.

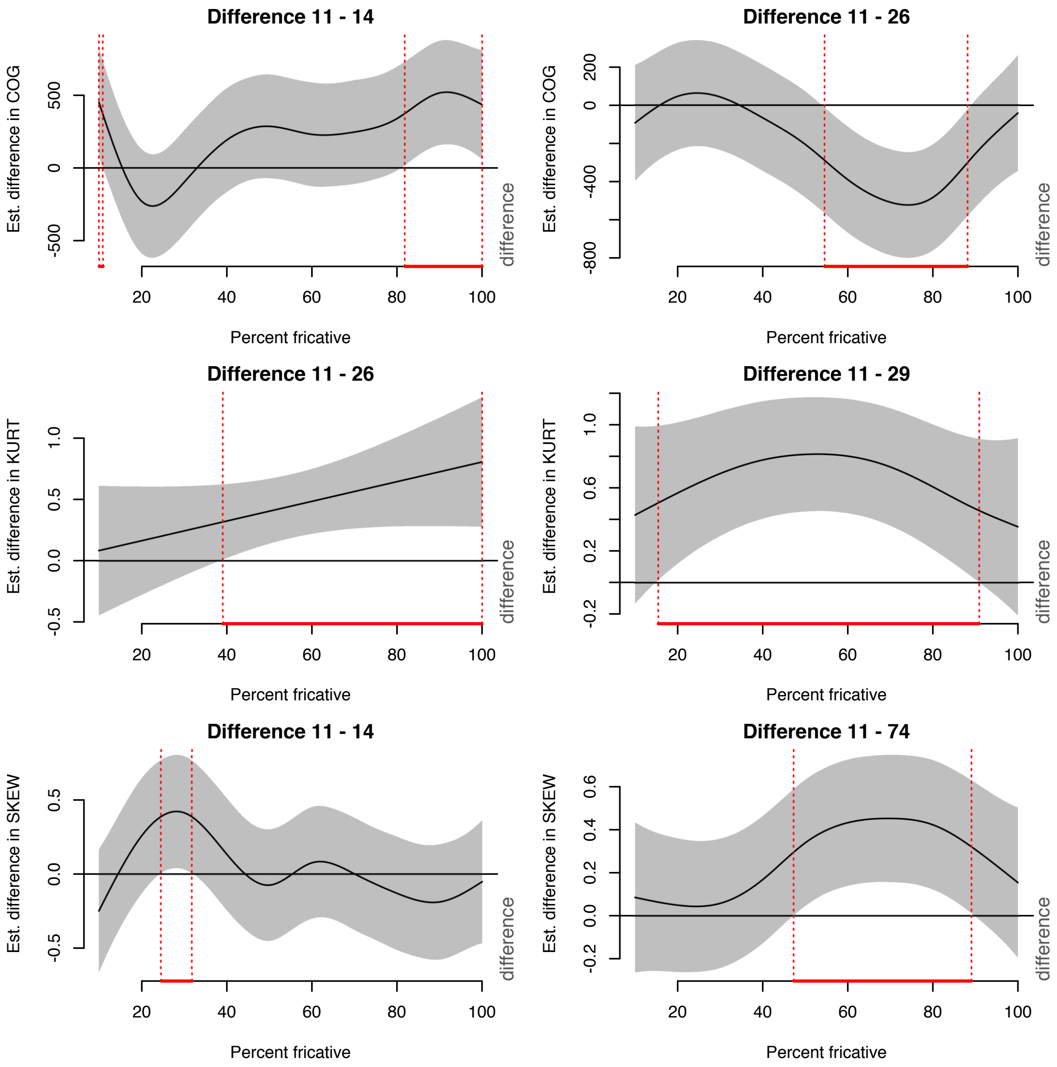

Figure S1. Pairwise difference smooths in COG, kurtosis, and skew between z_{11} and other two variables for models in Figure 17 (in the paper).

REFERENCES

Baayen, R. H., van Rij, J., de Cat, C., and Wood, S. N. (2016). Autocorrelated errors in experimental data in the language sciences: Some solutions offered by Generalized Additive Mixed Models. *arXiv e-prints*, arXiv:1601.02043

Lenth, R. (2018). *emmeans: Estimated Marginal Means, aka Least-Squares Means*. R package version 1.3.0

Liaw, A. and Wiener, M. (2002). Classification and regression by randomForest. *R News* 2, 18–22

Simon, N., Friedman, J., Hastie, T., and Tibshirani, R. (2011). Regularization paths for cox's proportional hazards model via coordinate descent. *Journal of Statistical Software* 39, 1–13. doi:10.18637/jss.v039.i05

Sóskuthy, M. (2017). Generalised additive mixed models for dynamic analysis in linguistics: a practical introduction. *arXiv e-prints*, arXiv:1703.05339

Wood, S. N. (2011). Fast stable restricted maximum likelihood and marginal likelihood estimation of semiparametric generalized linear models. *Journal of the Royal Statistical Society (B)* 73, 3–36. doi:10.1111/j.1467-9868.2010.00749.x
